# Health and Disease

**Published:** 1881-09

**Authors:** 


### HEALTH AND DISEASE.

And so, although the thermometer was about twenty above zero, and a strong cold west wind was blowing, we went to work, and when it was completed, the whole body was in a profuse perspiration; but the neck, throat, and upper part of the chest felt as if they were frozen a foot, more or less, deep; and then came out the expression, with very considerable unction, "What an unmitigated fool have I been, to be working all this time with head, and neck, and chest exposed to such a piercing cold wind!" Then came up visions of croup, diptheria, quinzy, putrid sore throat, and a dozen other hobgoblins, any one of which might have put us past cure within forty-eight hours. The only course to pursue was to keep up a vigorous circulation, so as to prevent chilliness; for if that had taken place, pneumonia would have been a pretty certain event; but exercise for this purpose was out of the question, because we were already jaded out; perhaps a non-te-totaller would have resorted to a deep and hearty swig of wiskey; but that was not in our line, and besides there was not a drop in the house, then there remained but one means left, the application of heat, by means of fire and hot water; so we paddled the hands and face in water as hot as could be borne, and it was exceedingly grateful; then dressed as rapidly as possible and sat by a red-hot kitchen range, until the whole body was perspiring again; took breakfast, and went to writing an article for the Journal about murdering children in the public schools. As far as we know, we did not suffer the slightest injury by the unwise exposures which have just been detailed. Perhaps the first thought of the reader would have been to have applied the hot water to the neck, throat and upper part of the chest; that would have been inconvenient, would have been but partial, and would have dribbled the upper end of the flannel shirt, which was already wet with perspiration and was also very cold, but the hands and the face were at the extremities, and hot water would bring the blood to them, through the parts which seemed to be so cold, and thus warm them up in its progress; the hands could be kept continously hot, by keeping them immersed in hot water; the same with the face; but to apply water to the neck, with the hand or sponge, would be but for an instant, and only for the space which was covered by the hand, and it may be useful to remark here, that the safest way to cool off, even in summer, is to dip the hands in hot water and raise them in the air; do this successively; the philosophy of it is, that the evaporation of heat is very rapid, and is carried off by the steam caused by the hot water and warm skin; cold water will do the same thing, because the heat of the skin converts it into steam, but it is a harsh method, and is never so safe; and many times, when a person comes into the house from a cold walk or ride, feeling as if a chill were iminent, a most comfortable method of averting the chill, and of warming up the whole blood, is to immerse the hands in hot water, and keep them in motion; for if allowed to remain still, the layer of water next to them becomes in a measure cold; while by moving them about, the skin finds new layers of hot water, and thus every motion is grateful. The same may be said as to the feet; it is certainly a very agreeably method of getting warmed up; and when you have become so, it is well to dip both hands and feet into a vessel of cold water, for a second or two. Of course, if the extremities are frozen, stiff with cold, or without feeling, then snow or very cold water should be applied under the supervision of a physician.

### Mental Rest.

No man can study advantageously all the time; there must be some relaxation, or the brain will become disorganized, or otherwise hopelessly diseased. No man ought to study hard on one subject more than four hours a day, and give ten hours to purposes of dressing, sleeping and eating, leaving ten hours for mental recreation, that is, mental rest, which is done in two ways: first, engaging the mind in thinking about something else; that is, putting other organs of the brain to work; second, engaging in muscular motion, which does good in two ways; it works out of the system the waste particles made by hard thought, and thus purifies the blood, fitting it for building up the brain, repairing its wastes, making it ready for new work. But muscular motion does good in another way; the mind is diverted to it, and is thus rested from the main study; hence the true policy of hard students is not to sit, or loll, or lounge about, but to be doing something with the hands or feet which is of sufficient interest to engage the mental notice, and, if pleasurably engaged, so much the better, even by fifty or a hundred fold.

It will be useful to remark here that we do not like to use our eyes in reading or writing until breakfast has been eaten, because they are stronger all the day afterwards; nor do we use them in that way after twilight; this has been our uniform habit, with the result, as we think, that we were able to do without the use of glasses for eight or ten years after our old school-mates of the same age had been using them, and we are writing now without any glasses whatever. Albert Barnes, the eminent commentator, rose habitually at four o'clock for study, and soon had to abandon all study, go abroad, and, after years of lost time, prematurely laid on the shelf from diseased eyes.

Literary and professional men, in consulting us, often inquire, with great earnestness, how can we take exercise in a large city, or town or village—"there is nothing that we can do." In the first place, eat about one-half less every day, and you will at once require but half the amount of exercise.

Literary and professional men maintain an idle theory when they consider every moment lost which is not employed in reading, writing, or investigation; it is loss of time in the long run which should alarm the individual; it is the curtailment of human life for ten, twenty, and even thirty years, which should startle the mind and lead to a wiser way of life. Whoever indulges in brain-work over four hours a day habitually, does in proportion shorten his life, or at least shortens the term of his usefulness; this is a great general rule, to which there may be some exceptions; but let the reader take it for granted that he is not one of those exceptions; on the contrary, whatever of time spent in muscular activities, beyond the four hours of brain work, adds that much to the probabilities of a longer life, and a life, too, of greater efficiency.

### Brain and Body Work.

Physiologists, after patient and close inquiry, have arrived at the important and practical conclusion that the power of the entire man, his vitality, is as much expended by two hours of deep mental effort as by a whole day of ordinary bodily labor; this fact seems to be founded on observed physiological laws; hence, the man who spends four hours in the twenty-four in earnest mental labor, goes to the utmost allowable limit for a day's work, and all the time that remains, after deducting ten hours for eating, sleeping and dressing, should be conscientiously expended in muscular exercises which require no special brain effort, and such exercises should always, by preference, be those which are agreeable, useful and profitable; for they not only promote the healthful condition of the body, but give rest to the brain, which, by that rest, recuperates its powers; many can remember, when turning back to their school-days, that they have gone to bed feeling that they did not know their lessons, yet, on rising in the morning, the mind would run over them with a gratifying and surprising clearness. It is this which accounts for the observation that persons have striven hard to remember some important fact, or as to where valuable papers have been laid, and towards morning, when the mind began to awake, a little before the body, this being the time of dreams, the point is made clear in the form of a dream, thus showing that rest of the brain, whether by actual sleep or the passive, comparative rest which manual labor affords, gives mental activity, vigor, perspicacity; from these it follows that no form of muscular exercise is ignoble in a student, a brain-worker, which has to be done by some one, and by being done by him, will save money, or will save the time of another, who, perhaps, may already be over-taxed. How many servants are over-taxed! how many faithful, uncomplaining wives are over-taxed! and sons and daughters sometimes; and clerks, and apprentices, and other employees. In every dwelling in a large city, there are many things which the master could do which would reflect benefit on himself and others

Frosty Weather.—Few have failed to observe what a vigor and elasticity are imparted to both mind and body by a frosty atmosphere, and what a loss of all these there is in a hot summer day; this is probably owing to the fact that at noon of any clear frosty day in winter, there is ten times as much elasticity in the air as there is at any noon of summer; hence to all invalids, the days most valuable for exercice are those of frosty weather, and those least beneficial are where it is warm or thundery; hence every hour of daylight spent in the open air in frosty weather in some kind of out-door activities is that much gain to the vitality of the system, imparting vigor to the mind, elasticity to the body, and elevation to the moral feelings and power of the man.

Life Lengthened.—In all countries and all latitudes, the well-to-do live longer than the poor by an average of eleven years; this shows the deleterious influence of an anxious mind on the bodily health, the anxiety for to morrow's bread. Pensioned persons live indefinitely long; the poor-houses of Great Britain can any day turn out a large army of men and women among the eighties and nineties who have been in those institutions for twenty and thirty years, owing in great part to an habitual feeling of confidence that ample provision is made for the future, and the mind is at rest; but it must not be forgotten that the cleanliness, the plain food and the regular habits, compulsory in those institutions contribute greatly to the same end. Insurance companies are ingeniously making use of these facts, and doubtless with some show of reason, for certainly a man feels greatly more at ease in his mind when he feels assured that in case of his death, a handsome amount of money would certainly be paid over to his family, than if the blighting and depressing impression were all the time weighing upon his spirits that his death would leave them in destitution. A British medical journal says that in the eighteenth annual report of the Providential Assurance Co., it is shown that for three years in succession, the rate of mortality was twenty-three per thousand among the unassured, while it was twenty-one per thousand among the same class and occupation of persons who kept up their assurance. It is beyond all question that a quiet mind contributes to the health and well-being of the body. In this connection it may be stated that

Outbursts of Passion do certainly tend to shorten life, from the disturbing influence which they have on the circulation, for nature loves regularity and equanimity; this important truth is beautifully demonstrated in the ascertained fact that members of the Society of Friends throughout England, live, on average, twelve years longer than others in the same stations and callings in life.

### Sea Sickness

Is caused in great part by the confusing effect which the tossing on the water has upon the brain, and multitudes of ways have been pursued for avoiding or at least mitigating this annoyance. The best plan is to let it have its course and rid the system of that excess of bile which is almost always present in this over-eating age ; the general health rarely fails to be greatly improved by it, although in very rare cases, perhaps not over one in a million, dies under the effects of the long continued and exhausting retching. If a person will lie down with the eyes closed, and not allow the head for an instant to be raised from the pillow there is an almost entire exemption from nausea and other discomforts, but the result of this course is that it will be necessary to keep a-bed during the entire voyage ; the effort should be to shorten the sickness and get rid of it as soon as possible, and this is best done by not lying down at all, but resolutely keeping on the feet on deck, in the open air, if the weather permits, that is, if it is not raining; this requires moral courage and some considerable force of will and character, but it seldom fails to abridge the period of sea-sickness, sometimes to confine it to a few hours duration and then the remainder of the voyage can be enjoyed as it ought to be.

The tendency to nausea on ship-board is abated somewhat by any stimulus which acts decidedly on the nervous system, such as chloroform, brandy, opiates, etc. Irritants, such as the strongest spices, abate nausea; so will great mental emotions, in short, any thing which draws off the attention of the mind. No person can get sea-sick if the ship is on fire, nor will a person who is drunk. A brisk purgative is good just before going on board or a dose of medicine taken the night before. Still the wisest, most healthful and most expeditious method of meeting sea-sickness is to avoid all preventitives, all medicines, and manfully determine to keep upon your feet and let it do its worst.

In this connection space may be given to

### Sea Voyages, and the best means of enjoying them; and first of all have a plenty of woolen clothing and wear it even in midsummer except during the middle of still days ; but every day, and all day a good flannel shirt should be worn next the skin even in the tropics, to counteract the baleful effect of damps, fogs, and changes of temperature. The British government compels its sailors to wear woolen flannel shirts all the year round in all latitudes as a result of its observed necessity in keeping off disabling diseases.

PROTECTING the FEET

from the dampness of the decks is an indispensable item of health and comfort on ship-board as the boards are seldom dry for two hours at a time in any voyage. Thick soled shoes and woolen hose should be worn at all times while at sea. Much has been said and written in praise of the

PURE AIR OF THE SEA, but as a matter of fact very little of it is obtained by passengers as a general rule, because a bilgy odor pervades the cleanest ship's cabin, and when it is taken into account that in these cabins passengers confine themselves from sun down to a late breakfast next day, and that soon after breakfast the decks, having been washed, are still wet, making it near noon before it is safe for ladies, with their thin shoes, to promenade; it is evident that a very few hours of the most pleasant days are devoted to the breathing of the pure salt sea air, and when it is remembered too, how few days at sea have an entire exemption from rain and raw winds, it is evident the much lauded good effects of sea voyages, especially to invalids, is more a myth than a r[illegible]. The truth is, to obtain the very highest healthful advantages of pure air, nothing approaches moderate, leisure working in

THE GARDEN OR THE ORCHARD.

Next to that, especially for the ailing, is a continuous

HORSEBACK JOURNEY, every day, rain or shine, with some exhilerating object a-head, other than the mere healthfulness of it; in fact, no form of exercise can promise any marked good effect unless there is some other motive than that of health to give vivacity to the mind, and elasticity to the body, the spirits and the circulation.

GLYCERINE,

As an article of food, as a nutrient, is well worthy of being brought into public notice. Sweet oil in Palestine and other old countries, has for ages been used as an article of daily food, and glycerine may be considered as the essence of pure "sweet", that is "olive oil," they being one and the same ; it is a perfectly neutral and bland fluid, and the most penetrating perhaps in all nature. Oil itself will permeate where water will not ; and glycerine, which may be considered the ethereal part of oil, has this property to a most remarkable degree ; it penetrates the solid bone ; if poured into a mixture of blood and matter, such as is expectorated from consumptive lungs, it will get in between the globules of each and show them with great distinctness ; being thus penetrating, it is the very best application for all feverish sores, for inflamed or dry surfaces simply from its quality of penetration and want of evaporatability ; the first and highest value of any poultice is its capability of keeping moist for the longest time ; no one ever thinks of a dry poultice ; glycerine keeps a part moist longer than any substance known, hence its value as above, mixed with an innoxious dry powder, called sub-nitrate of Bismuth, so as to make a thin paste or poultice. It is one of the very best applications known for

BURNS, whether in children or adults, giving an almost instantaneous relief from suffering, by its entire exclusion of the air and by its moistening, hence cooling, soothing effects, promotes a speedy healing process, always safe, simple and efficient. A few cents will buy half a pound of it at any good drug store, and every family should have some at hand, in a bottle, plainly labelled, with a bottle of glycerine at its side.

If glycerine alone is applied with a common brush to the surface of the throat in that dangerous malady dyptheria, in a few minutes its permeative quality enables it to sink into and between the molecules of the false membrane, dissolving and than detaching it, in a few hours ; so also in that other most alarming malady, the croup of little children, as it not only penetrates and thus disintegrates, spreads apart the tissues of the false membrane, but when it gets to the bottom of it, it spreads out between the layer of the membrane and the more natural mucous surfaces beneath and thus not only detaches the membrane, but imparts a healing, soothing influence to the inflamed condition of the natural surfaces. Hence also if applied to

### Dry Sores, its affinity for organic globules is such that it seems to be attracted to them through the dry scab, moistens it, detaches it, and causes a new and healthful surface to grow beneath it; it has the same delightful, soothing and healing effects when applied to

### Blistered Skin, taking out the inflammation, cooling the parts and restoring them to their natural condition.

### Painful Sores are very gratefully and speedily relieved by a mixture of morphine and glycerine. But more striking benefits are observed in its use as food, from its inherent nutritious qualities in all that large class of cases where a person needs

### Food Rather Than Physic.

As evidence of its capacity to fatten children a physician states in the American Journal of Pharmacy, its chemical formula being six parts carbon, seven parts hydrogen and five parts oxygen,

1st. An infant six months old, recovering from a severe diarrhœa, kept quite emanciated and pale. Glycerine was ordered for it, and in a few days a change was remarked in its appearance for the better, and in four weeks it weighed eight pounds heavier. 2. A child, sixteen months old, had its head covered with one continous scab,—porrigo. This was a family complaint, and resisted all manner of treatment for a very long time in all the other children. In the above case I resorted to glycerine, both internally and externally. A cure was effected in three months. 3. A girl, seven years old, recovering from measles, retained her cough, emaciation, and nervous irritability. Dullness over apex of left lung; roughened breathing. No doubt the case was chronic pneumonia. Glycerine, as a last resort, was ordered in teaspoonful doses in water, three times a day. Recovery in six weeks. 4. A

strumous boy, much emaciated, had hacking cough and night sweats. Pulse frequent. Sleep disturbed. Abdomen tumid and enlarged. Cervical glands swollen. Bowels irregular. Fecal discharges clay-colored. His case was such, that no one expected any more than a partial palliation. After other treatments had failed, I ordered glycerine in tea-spoonful doses, in which were dissolved four grains of ferri ammon. cit., and one half of a grain of quinia, four times a day. This he continued for a year, and was in remarkably good health three years after.

### Mineral Waters.

"This is like the water of twenty-five years ago," said an habitue of Saratoga Springs the other day as he quaffed his glass at the fountain.

It is a well known fact that the powers of the Saratoga waters have diminished greatly of late years in consequence of the fountains having been tampered with, in order to promote a more copious flow of the "health-giving liquids." By a very ingenious device all owners can present to those who frequent the springs as well as those who use the water at their own homes, the pure, unadulterated article as it is found in its natural state fifty feet beneath the surface of the soil; it is at that distance the bottles are filled and corked, without any possibility of admixture with the external air or the escapement of their essential virtues; these waters are furnished in their natural state, hence with all their natural virtues. Beyond all question, the best water for a man to drink, in health or disease, is that which is the purest as it comes from its natural fountain. Whatever healthful effects result from the drinking of any impregnated waters is in proportion to the increased action given to the bowels, the kidneys and the skin; by acting on the intestines as mineral salt waters do they expel the excrements of digestion, that is the solid refuse of the food, after the nutritious portions have been extracted and applied to the purposes of sustenance and life. Sulphur waters are known to exert a decided influence on the skin, relaxes it, opens its pores, and allows the passing out of the system a large amount of its impurities, especially the useless portions of the products of combustion of vegetable food in particular, but the waste products of the combustion of animal food are carried away through the kidneys and such waters as act upon them are the most useful in this class of cases, and physicians as well as individuals should remember that the value of all mineral waters depend upon their applicability, in these three forms, and should act accordingly.

### In-Growing Toe Nails are a source of excessive discomfort and sometimes of almost insufferable pain ; formerly the savage mode of treatment was to take a pair of pincers and drag the whole nail out, but now a prompt and painless cure may be effected simply by inserting the dry ses-qui-chloride of iron between the nail and the flesh and powdering the latter with it also, then apply a dry bandage, and a cure follows after two or three applications, a day or two apart.

### Shoes.

The feet are the great avenues of death to multitudes every year ; cold feet, damp feet, wet feet give colds which settle on the lungs and light up the fires of consumption which burn away until nothing is left but skin and bone, and the poor body falls into the grave, hence the importance of clothing the feet properly. More than two thousand years ago the Jews made shoes of leather and wood while their soldiers sometimes formed them of brass and iron ; the Egyptians used papyrus ; the Chinese wore shoes made of silk, leather, rushes, iron, brass, wood, bark, gold and silver. The Greeks and Romans used leather, reaching generally to the middle of the leg, sometimes however using only enough leather to cover the sole of the foot, black shoes were worn by ordinary persons, of rank, the women wore white, but on ceremonial days, the magistrates wore red shoes. The ingenuity of modern times has not as yet furnished a convenient, comfortable and healthful covering for the feet ; the requisites are pliancy so as to allow the toes, nails and joints to maintain their perfectly natural condition, which so greatly promotes easy and healthful walking ; the next necessity is that the soles shall be impervious from dampness without and dampness from within ; there should be ample ventilation, otherwise the gases and perspiration constantly escaping from the feet, as well as from every part of the human body will condense, not only causing an unhealthful dampness, but so confining the odors as to sensibly affect the atmosphere of a large room, the moment the covering is removed. The shoe itself should not come above the ankle, because, first, the emanations would readily escape, and fresh air be constantly admitted; second, because the ankle, being unsupported, would by use, more rely upon itself, would become daily stronger, more supple and more elastic, with greatly less danger of dislocation; hence the wearing of boots habitually is a gross absurdity, a relic of barbarism. The sole of the shoe should be painted several times with castor oil, being allowed to dry most thoroughly between each application; this would take the disagreeable creak out of shoes and would keep out the water very effectually without the necessity of having a cumbrously thick sole. The upper part of the shoe should be made of canvass, of a color suited to the weather and to the habits of the individual, fitting but moderatly tight. The feet should be placed in a basin of cold water every morning for a few seconds, just deep enough to cover the toes; wipe dry, dress and walk off. Once or twice a week the feet should be held in water, made comfortably warm, for some ten minutes, adding hotter water from time to time, using a little soap; if at the end of this bathing at night the feet were placed in a pan of cold water, toe-deep for less than a quarter of a minute, it would greatly aid in giving tone to the skin, vigor to the circulation and softness to the skin, and thus do much towards keeping them comfortably warm. A tablespoonful of Chloride of lime in a basin of warm water is an excellent wash for removing foot odor: before retiring to bed most especially in fire time of year, hold both feet before a blazing fire, stockings removed, for ten minutes at least, rubbing them with the hands all the time until they feel perfectly dry and warm; such a process will warm the feet more effectually in five minutes than can be done in an hour by holding them to the fire with stockings and shoes on.

### Sometimes, without apparent cause, a person will suddenly wake up to the knowledge that his feet are cold and a disagreeable sensation is caused which pervades the whole body and the mind and temper become fretful and morose; this is often the case in the very midst of summer; when this is observed you are taking cold and you should instantly treat the feet to a blazing fire as named above; if this is not practicable, give them a hot foot bath as just directed; in either case you will not only avert the cold but you will experience a feeling of comfortableness, which is absolutely delightful; this same kind of bath is the speediest and most comfortable means of warming the feet when they are found to be uncomfortable cold after coming in from a walk, or a long day's work.

Many ways have been devised for rendering the upper leather of shoes impervious to water; a much better plan is to keep out of the water, for whatever will keep water out will also keep the perspiration and ill odor always in; some persons' feet are smelled a mile off, more or less. To make leather impervious, is to make it board like, hard, unyielding, and hot as fire of a summer's day; but if it be absolutely necessary at any time to wear a shoe which shall exclude water, the application of castor oil or petroleum with a brush and then allow it to dry, is perhaps the most familiar, accessible and facile mode, known. If the space between the upper leather and the sole of pegged shoes is occasionally dressed with petroleum, until the pegs are saturated with the liquid, there will be no ripping.

### Hands.

The hands are as much abused as the feet, and to know how to serve them properly in winter time may be a convenience and comfort for many. It is a great luxury to go to bed with clean hands, washing them in soap and warm water in winter, so as to dissolve the dust and dirt which settles in the crevices and fills up the pores, thus leaving them soft and pliable; this should be done also in the morning before washing the face, care being taken to rinse them of every particle of soap with clean pure water, and most thoroughly dry them after washing with a soft cloth; otherwise, the skin will soon chap and crack open, especially if gloves are not put on when the fire is stirred or approached for any domestic purpose. The hands should be put in water as seldom as possible in cold weather and if immediately after the washing a teaspoonfull of spirits of wine were rubbed into them or a few drops of honey—the alcohol is preferable—it would greatly tend to prevent chapping altogether. In coming down stairs of a cold day the bannister should not be touched with the hand unless a glove is worn ; if that is not convenient, let the cuff of the sleeve be moved along the bannister if support and guidance is needed in descending the stairs; this may seem a small matter but a lady does not well like having a hand as course as a corn-cob when she holds it out to give her friends a New Year's greeting; besides, chapped hands interfere greatly with domestic requirements, for to say nothing of the painfulness sometimes attending it, it is often impossible to knit or sew with any kind of comfort or convenience. Cold cream, as it is called, tends to prevent and cure chapped hands; and for the convenience of our lady readers who live in the country remote from drug stores, the following directions are appended:

Take of oil of sweet almonds three ounces and a half, one ounce of spermaceti, one hundred and twenty grains of white wax and two ounces or four tablespoonfulls of rosewater; melt the oil, spermaceti and wax in a water-bath, that is in a vessel set in another vessel of boiling water, as a cabinet-makers glue-pot; then add the rose-water gradually, stir it up with a clean stick until it is pretty well cooled, pour it in a tea-cup or other delf ware and rub it into the hands after each washing. Badly chapped hands improve rapidly, if after the night washing common sweet oil is rubbed in thoroughly and a pair of old kid gloves are worn during the night for several nights in succession.

### Warts.

will disappear from the hands in a few days if red ash bark is boiled in water, strong, and the liquid applied several times every day; or if a bit of wood, an old match is dipped in aquafortis or nitric acid and rubbed on the head of the wart night and morning, it will soon disappear. Great discomfort sometimes arises from the skin at the body end of the finger nail getting shiny and then turning up; this is occasioned by the skin attaching itself to the nail and being drawn out by the nail as it grows forward, until the skin snaps by the extension, causing sometimes severe inflammation; the best prevention is to take once a week a wooden or iron paper cutter, or even the thumb nail of the other hand and push back the skin; if a knife is used, it will make a white spot on the nail, unless done with great care; ladies sometimes prevent this by pushing back the skin with a soft cloth every time the hand is washed.

In the days of Louis XIV. the ladies of the court as a means of beautifying the skin of the face and hands and keeping it soft and velvety always washed in alcohol; perhaps pure warm water answers as good a purpose; cold water has a tendency to harden the skin, to make it coarse and certainly is not as good a cleanser as warm water.

CORNS.

are the botheration of the vast number of silly folk, who for appearance's sake, render themselves liable to purgatoric suffering during the entire space of their natural life thereafter; there are multitudes of cures for corns, the very quickest and most certain method known to us, is to cut off the toe, and be done with it; a better plan, to our own personal knowledge, in reference to all corns, is not to have any; and this exemption can be secured by wearing shoes which can be drawn on the feet without much straining with two pair of woollen stockings, then when you get home pull off one pair and wear the shoe. But, as some poet has written, that variety is the spice of life, various "infallible" cures for corns are appended for the amusement and practice of our afflicted reader.

"Pare the corn as close as you can, then get a thin piece of India rubber cloth, about the twentieth of an inch thick, (the pure India rubber is the best, but that made of cotton will do) and where the corn is on one of the toes, make a stall of it; or where it is on another part of the foot, sew it on the inside of the stocking and large enough to cover the corn well. By continuing the application from four to six weeks, and paring the corn as the callous skin loosens, the corn will disappear. The application of the rubber will give immediate relief to the pain. The principle of the cure is to assist nature in restoring the skin to its natural condition again."

One objection to the above is, it is too tedious and troublesome; another more serious is that a number of cases are recorded in medical works where the paring of a corn has been followed by a fatal bleeding or inflammation, irritation, lockjaw and an agonizing death.

—Scrape a piece of common chalk, put a small portion of it upon the corn and bind it with a linen rag. Repeat the application for a few days, and you will find the corn come off like a shell, and perfectly cured. The cure is simple and efficious.

—Mr. Wakely, in the Royal Free Hospital, London, is in the habit of applying glycerina to corns. It softens those excrescences so that they may be scooped out with ease.

—Take a piece of lemon, nick it so as to let in the toe with the corn, tie this at night so that it cannot move, and you will find the next morning that, with a blunt knife, the corn will come away to a great extent.

OUR METHOD.

The safest, the most accessible, and the most efficient cure of a corn on the toe, is to double a piece of thick soft buckskin, cut a hole in it large enough to receive the corn, and bind it around the toe. If, in addition to this, the foot is soaked in warm water for five or more minutes every morning and night, and a few drops of sweet or other oily substance are patiently robbed in on the end after the soaking, the corn will almost infallibly become loose enough in a few days to be easily picked out with a finger nail; this saves the necessity of paring the corn, which operation has sometimes been followed with painful and dangerous symptoms. If the corn becomes inconvenient again, repeat the process at once.

